# Extrinsic pulmonary artery compression mimicking acute pulmonary embolism

**DOI:** 10.1002/ccr3.1099

**Published:** 2017-08-17

**Authors:** Jonathan Xinguo Fang, Benjamin Xinhao Fang, Cheung‐Chi Simon Lam, Pak‐Hei Chan, Frankie Choi‐Cheung Tam, Chung‐Wah Siu

**Affiliations:** ^1^ Division of Cardiology Department of Medicine The University of Hong Kong Hong Kong SAR China

**Keywords:** Electrocardiogram in acute pulmonary embolism, extrinsic pulmonary artery compression, lung cancer, right heart strain

## Abstract

Right ventricular strain patterns on electrocardiogram such as right axis derivation and S1Q3T3 are well known for their diagnostic value in cases of acute pulmonary embolism. Nonetheless, these changes are not pathognomonic. We report a patient with electrocardiographic evidence of right ventricular strain secondary to an unusual etiology.

## Case History

**Figure 1:**
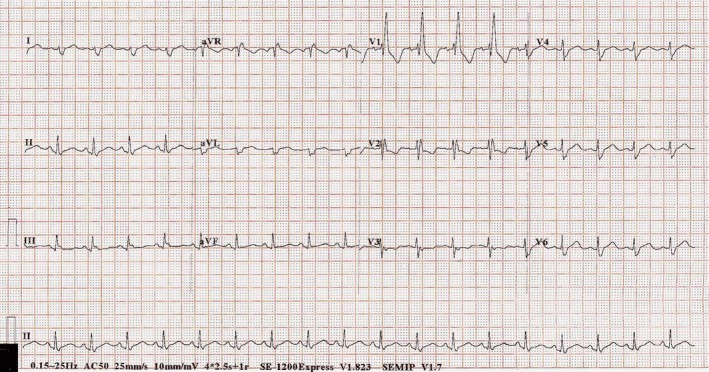
ECG at presentation.

**Figure 2:**
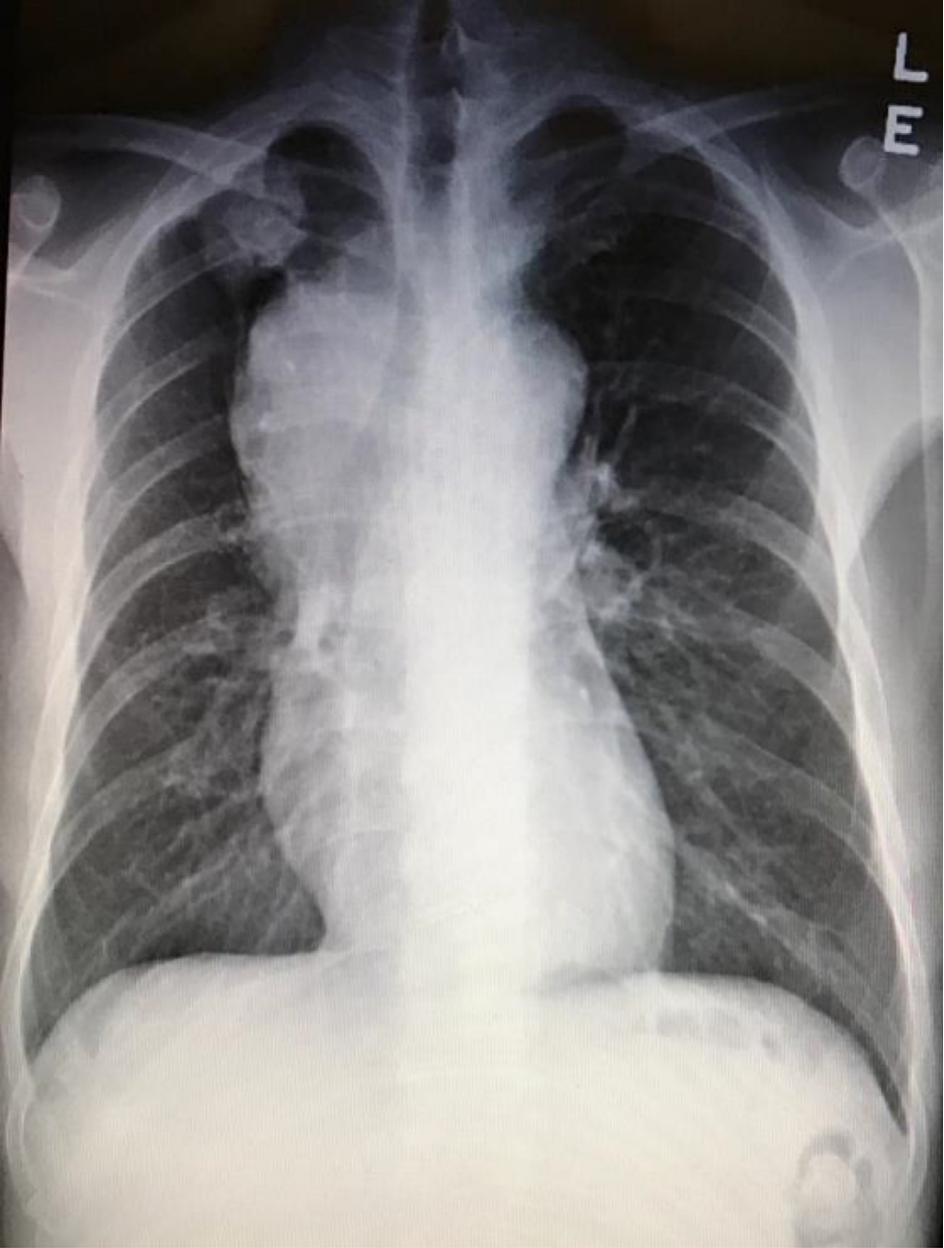
Chest radiograph at presentation.

**Figure 3:**
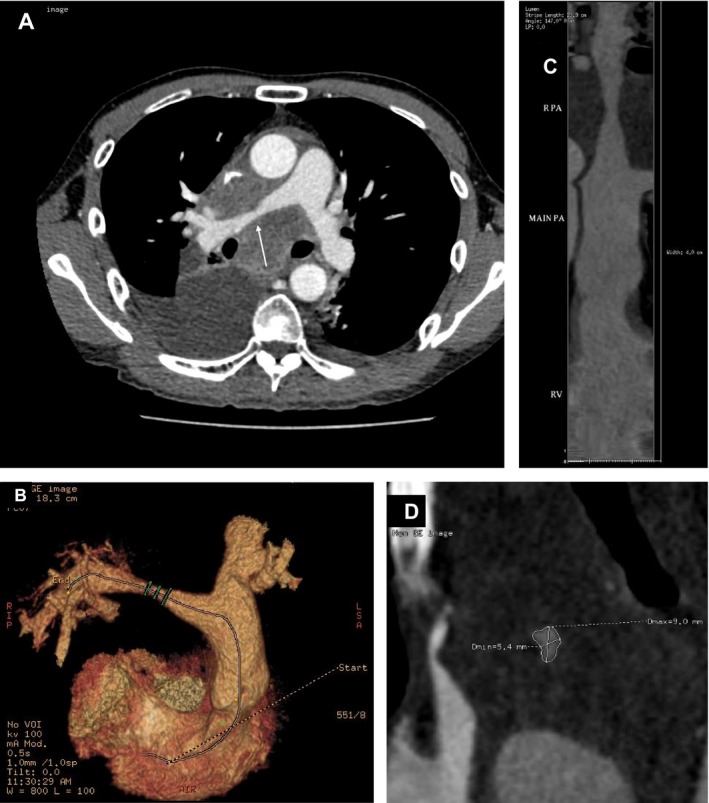
(A) Computer tomography with contrast of the thorax showing encasement with extrinsic compression of the right distal main pulmonary artery by the mediastinal mass (white arrow). The superior vena cava was also compressed, and slitlike, (B) 3‐D computer tomography reconstruction of the pulmonary and left and right main pulmonary arteries, demonstrating the narrowing at the distal right main pulmonary artery, (C) luminal diameter of the narrowed right distal main pulmonary artery relative to that of the main pulmonary artery, and (D) the cross‐sectional dimensions of the narrowed segment of the distal right main pulmonary artery measured 9.0 × 5.4 mm.

A 45‐year‐old man presented with a one‐month history of progressive shortness of breath and bilateral ankle swelling. He had smoked for 30 pack‐years. Physical examination revealed tachycardia of 110 beats per minute and a blood pressure of 120/82 mmHg. The oxygen saturation was 95% on room air. His jugular venous pressure was elevated to the level of his earlobes with a giant CV wave pattern. Bilateral ankle edema was also noted. Precordial examination revealed a parasternal heave, and a grade two‐over‐six ejection systolic murmur at the pulmonary area. Electrocardiography (ECG) showed sinus tachycardia of 110 beats per minute, right bundle branch block, and right axis deviation, T wave inversion in leads V1‐V2, an rsS' pattern in lead I, qR pattern in lead III, and flattening of T wave in lead III (Fig. 1). Chest radiograph revealed a 3‐cm lung mass at the right apical region, widened mediastinum, and oligemic upper lung fields (Fig. 2). The overall clinical picture was suggestive of bronchogenic carcinoma complicated by acute pulmonary embolism. Intriguingly, computer tomography with contrast of the thorax did not reveal evidence of pulmonary embolism but a heterogeneous mediastinal mass measuring 11.6 cm in diameter encasing the right pulmonary artery leading to severe narrowing, and another lobulated soft tissue mass of 3.8 cm in the right upper lobe (Fig. 3). Echocardiogram revealed an elevated right ventricular systolic pressure of 50 mmHg, and a peak gradient of 15 mmHg across the pulmonary trunk. Bronchoscopic ultrasound‐guided biopsy of the right upper lung mass revealed a high‐grade neuroendocrine tumor. Treatment consisted of six cycles of chemotherapy with etoposide and cisplatin, and radiotherapy over 4 months with minimal reduction in the right upper lobe tumor or mediastinal mass. He experienced progressively worsening shortness of breath, bilateral ankle swelling, and easy fainting during postural change. His systolic blood pressure ranged from 90 to 100 mmHg with persistent sinus tachycardia >110 beats per minute. Catheter‐based pulmonary artery stenting for palliation was offered, but the patient refused given the overall poor prognosis.

Pulmonary artery compression is an uncommon complication of mediastinal malignancies such as lymphoma, teratoma and other germ cell tumors, thymoma, carcinoid tumor, bronchogenic carcinoma, pericardial sarcoma, as well as metastatic carcinoma 1, 2. Nonmalignant etiologies include thoracic aortic aneurysm 3 and a calcified pericardial ring encircling the atrio‐ventricular groove 4. Due to the similar hemodynamic mechanism, the clinical features of pulmonary artery compression may mimic acute pulmonary embolism 5, 6. Nonetheless, the correct diagnosis can readily be made with computerized tomography of the thorax. For malignant pulmonary artery compression, the treatment goal is primarily symptomatic relief 7. Intervention is usually considered when there is significant elevation of right ventricular pressure and/or right ventricular dysfunction, severe pulmonary regurgitation, or worsening hemodynamics on interval echocardiogram or catheterization. Catheter‐based pulmonary artery stenting was first reported in 1998 by Müller‐Hülsbeck and colleagues 8. A successful intervention for pulmonary artery stenosis is defined as a 50% increase in diameter, a 50% decrease in pressure gradient across the vessel, a 20% decrease in pulmonary‐to‐systemic pressure ratio, or a 20% increase in blood flow by ventilation–perfusion scan 9.

## Authorship

JXF (first author): writer of the manuscript and involved in the clinical management of the patient. BXF (second author): radiologist and involved in and interpreting and formatting the CT findings. CWS (senior author): supervisor and advisor, professor of cardiology, the University of Hong Kong, edited the manuscript, and involved in the clinical management of the patient. CCSL, PHC, FCCT: contributed to the clinical management of the patient and given advices on writing the manuscript.

## Conflict of Interests

None declared.
